# Thyroid Carcinoma Arising From Struma Ovarii at Adolescence: A Challenging Case With Favorable Outcome

**DOI:** 10.7759/cureus.47163

**Published:** 2023-10-16

**Authors:** Mohamed Al-Shammaa, Ahmed Abdlkadir, Dhuha Al-Adhami, Ali Jawad, Akram Al-Ibraheem

**Affiliations:** 1 Nuclear Medicine, Baghdad Radiotherapy and Nuclear Medicine Hospital, Baghdad, IRQ; 2 Nuclear Medicine and Positron Emission Tomography/Computed Tomography, King Hussein Cancer Center (KHCC), Amman, JOR; 3 Rheumatology, Royal London Hospital, London, GBR; 4 Medicine, University of Jordan, Amman, JOR

**Keywords:** fertility preserving surgery, pediatric mso, thyroid papillary carcinoma, pediatric imaging, malignant struma ovarii

## Abstract

Malignant struma ovarii (MSO) is a rare and aggressive ovarian tumor that mostly affects adults but can occur in adolescents. Prompt recognition, accurate diagnosis, and multidisciplinary management are essential for favorable outcomes. Herein, we report the youngest case of an 11-year-old girl with a large MSO. First, conventional imaging revealed a large left ovarian mass, leading to a left oophorectomy. Subsequently, histopathological examination confirmed papillary thyroid carcinoma within MSO. Thyroid and fertility-preserving surgery were chosen after multidisciplinary consultation. Despite challenges, the patient had a positive outcome with no recurrence after 36 months. Therefore, the adoption of less invasive surgical approaches and vigilant follow-up can achieve remission, but more research is needed to further enhance our understanding of the disease's risk stratification and optimal treatment strategies.

## Introduction

Struma ovarii is an extremely rare ovarian tumor, accounting for less than 1% of all ovarian tumors [1]. It typically exhibits a benign nature, with only 10% of cases reported as malignant struma ovarii (MSO), most commonly presenting as papillary thyroid carcinoma [2]. Despite being recognized in adults, pediatric MSO has never been acknowledged up until recently [3-5]. The signs and symptoms of MSO are varied and nonspecific, typically including abdominal pain and discomfort, abnormal vaginal bleeding, and menstrual irregularities [6].

Conventional imaging modalities are often carried out during the initial diagnostic workup. These include ultrasound (US), CT, and MRI. To date, there is no consensus about the optimal management plan for patients with MSO. Management is often pursued with a multidisciplinary approach and surgery for suspected ovarian tumors and incidental pathological diagnosis is usually definitive for MSO [7].

In the pediatric population, the management of MSO is even more challenging since thyroid and fertility-sparing surgical approaches must be prioritized in adolescent patients. In addition, many of these patients would require lengthy and diligent follow-up.

## Case presentation

**Figure 1:**
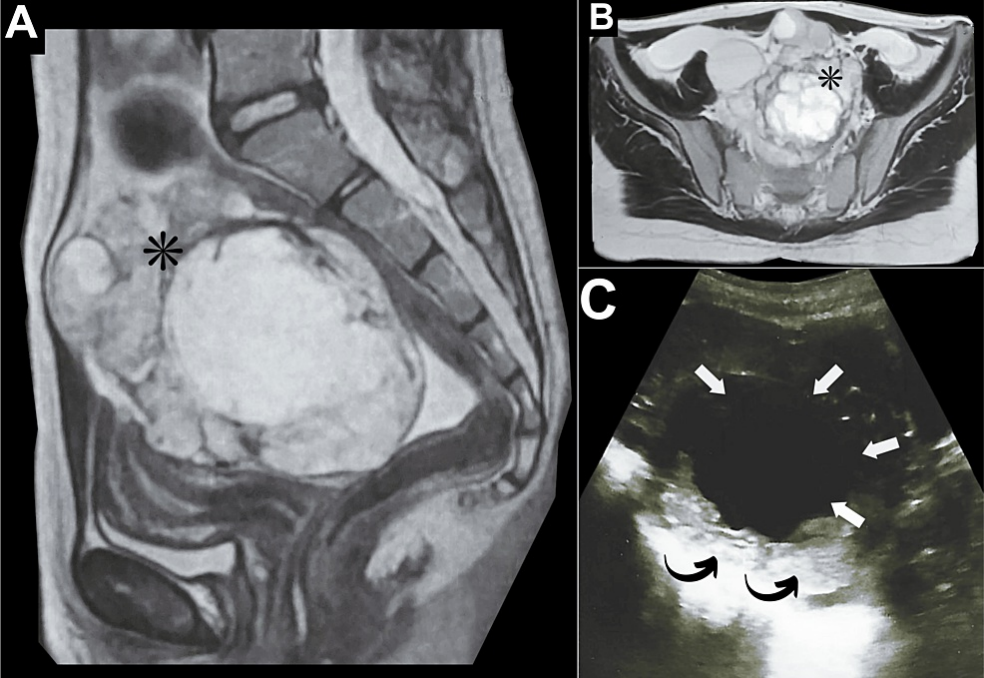
Left ovarian mass (initial diagnostic workup) (A) Initial sagittal T2 pelvic MRI revealed a large ill-defined complex multilocular lesion occupying the left ovary demonstrating contrast enhancement (asterisk). (B) Initial axial T2 MRI revealed a large, ill-defined, complex multilocular lesion (measuring approximately 13 × 8 cm) occupying the left ovary with a thick wall extending to the midline and into the right side after contrast and soft tissue enhancement (asterisk). (C) Initial pelvic US revealed a large left ovarian lesion (measuring approximately 9 × 7 cm) with thickened walls (curved arrows), suggesting a complex left ovarian cyst (arrows).

An 11-year-old female presented with left lower quadrant abdominal pain, which progressively worsened over a seven-month period, particularly during the last month of her presentation. The pain experienced was colicky in nature. It was aggravated by her menstrual cycle and radiated to her back. After seeking medical consultation, she underwent a gynecological evaluation. Upon physical examination, the patient was found to have left lower abdominal swelling and tenderness. Her laboratory results showed a hemoglobin level of 13.6 g/dL and a white blood cell count of 6,600/μL. In addition, the levels of beta-human chorionic gonadotropin and cancer antigen 125 (CA 125) were within the normal range. Therefore, conventional imaging modalities were performed (Figure 1).

**Figure 2:**
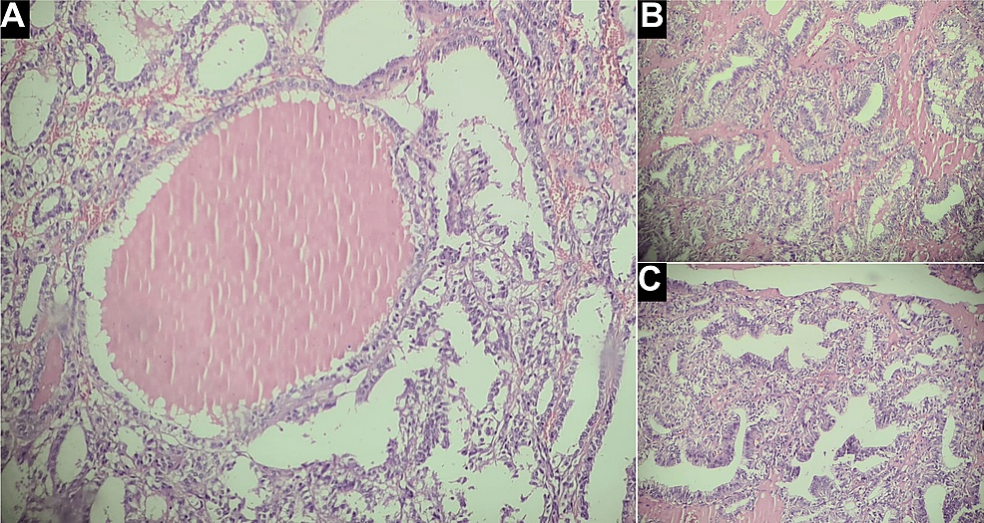
Left ovarian mass (histopathology) Histopathologic examination revealed (A) a large thyroid follicle filled with colloid, surrounded by follicles showing increased cellularity and nuclear alterations, (B) a predominant papillary architecture, and (C) malignant papillary arrangement with clear nuclei, overlapping nuclei, and focal calcifications.

A pelvic ultrasound revealed a substantial, large left adnexal mass (Figure 1C). This mass was observed to have thickened walls indicating a complex left ovarian cyst. Concurrently, an MRI of the pelvis demonstrated a giant multiloculated left ovarian mass (Figure 1A-1B). The mass was complex (cystic-solid) measuring approximately 13 × 8 cm in maximum dimension. In addition, a thick wall extending to the midline and into the right side demonstrating contrast enhancement was noted. Subsequently, the patient was referred for surgical intervention under the care of another senior specialist. A left oophorectomy was performed, and histopathological examination of the specimen revealed a large gelatinous mass measuring 14 × 8 cm, exhibiting features characteristic of papillary thyroid cancer within struma ovarii, including extensive thyroid tissue with multiple colloid-filled follicles (Figure 2).

**Figure 3:**
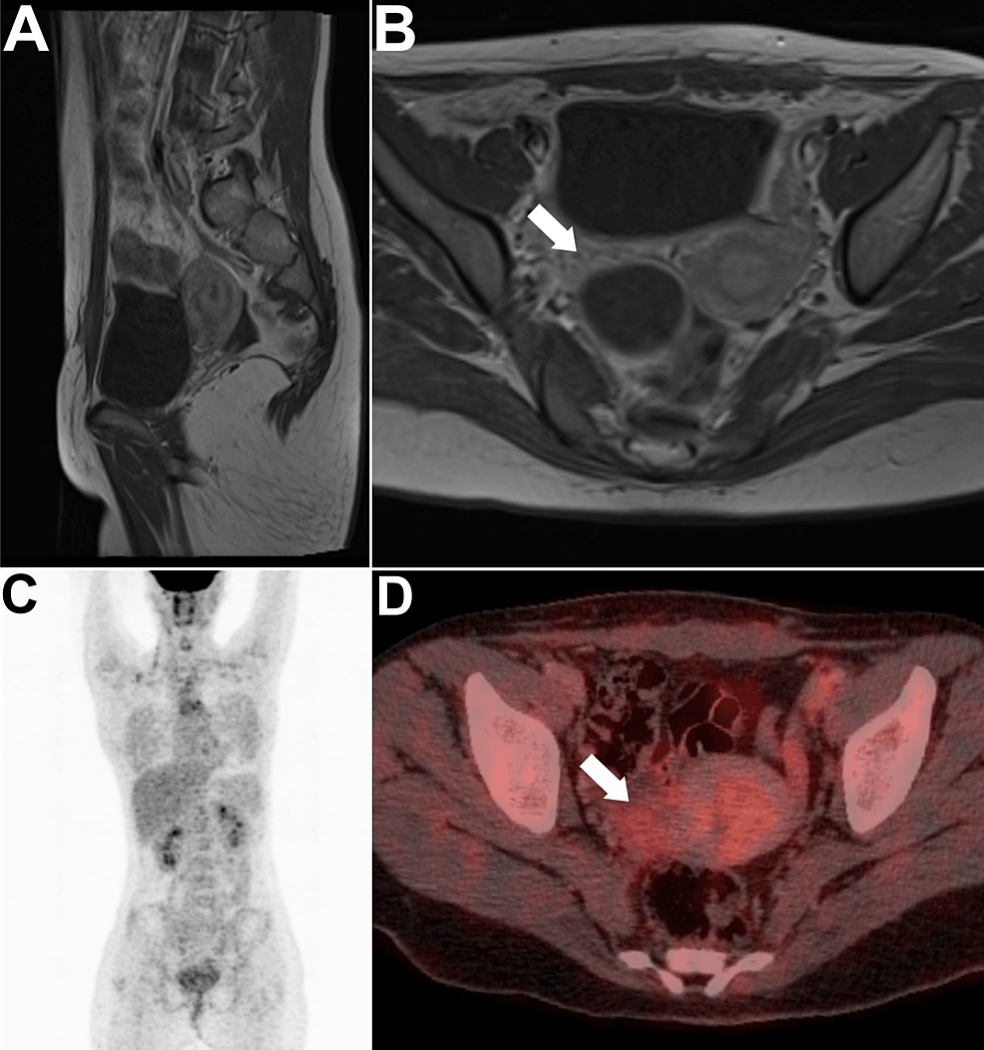
Diagnostic scans at the end of the third year following surgery (A) Sagittal T1 pelvic MRI revealed a clear surgical bed that was unremarkably normal. (B) Axial T1 MRI was also unremarkable. (C) Maximum intensity projection image rendered a normal scan. (D) Axial PET/CT image of the pelvis revealed a clear tumor bed that was unremarkably normal (arrows).

Postoperative thyroid function tests were within the normal range, and there was no clinical evidence of metastasis. The patient was diagnosed with stage Ia according to the International Federation of Obstetrics and Gynecology (FIGO) staging system [8]. Based on the decision of the multidisciplinary clinic (MDC), no additional adjuvant treatment was administered. Considering the absence of tumor extension in recent diagnostic modalities, it was decided to observe the patient closely for the next five years without pursuing total thyroidectomy, right oophorectomy, or radioactive iodine therapy. This approach aimed to preserve thyroid function and fertility, which are particularly important in this challenging age group. To date, the patient has maintained regular follow-ups consisting of routine pelvic US, neck US, and biochemical laboratory tests for thyroid function, serum thyroglobulin, thyroglobulin antibody, and CA 125. All aforementioned modalities were normal obviating the need for additional interventions. After three years of diligent follow-up, including comprehensive diagnostic assessments with pelvic MRI (Figure 3), the patient remained in good health with no signs of recurrence.

More recently, [18F]fluorodeoxyglucose positron emission tomography/CT ([18F]FDG PET/CT) was performed upon patient request in an outside cancer center for surveillance purposes and was unremarkably normal (Figure 3C-3D).

## Discussion

Pediatric MSO has been acknowledged in pediatric populations in the last three decades. To date, fewer than five cases of pediatric MSO have been reported [3-5]. Therefore, MSO can manifest across all age groups including pediatrics. There are a few possible explanations for the increasing incidence of MSO in pediatrics. One possibility is that improved diagnostic methods lead to more cases being identified. Another possibility is that environmental factors may increase the risk of developing MSO [9].

Most cases of pediatric MSO typically present with pelvic pain [3-5]. Other nonspecific gynecologic symptoms have also been reported. Although rare, some patients may also exhibit signs indicative of hyperthyroidism, such as weight loss, agitation, heat intolerance, tremors, and palpitations [3]. In these circumstances, nonspecific pelvic pain may follow symptoms of hyperthyroidism, warranting further investigation and imaging correlation [3]. Therefore, any nonspecific pelvic pain with a background of hyperthyroidism should warrant further diagnostic workup to exclude the possibility of having MSO. In regards to diagnosis, most patients are unexpectedly diagnosed with MSO after surgery for a pelvic or ovarian mass incidentally found on conventional imaging.

Conventional imaging modalities are helpful in detecting the MSO mass, depicting its extent, as well as for follow-up [10]. The usual US characteristics of MSO consist of a solid, heterogeneous mass with irregular boundaries. Additionally, the mass may exhibit small and scattered cystic components [11]. The solid segments of the mass can exhibit either hyperechogenicity or hypoechogenicity, and they may demonstrate increased vascularity on Doppler imaging [11]. The CT and MRI features of MSO are not always specific, and they can be similar to the features of other types of ovarian tumors [10]. However, the presence of a solid, heterogeneous mass with irregular borders is often a suggestive finding. In some cases, MSO may also present with hemorrhage, ascites, or lymphadenopathy [10].

In the pediatric population, the management of MSO is even more challenging as it must take into account fertility preservation for childbearing. However, this means that routine and diligent follow-up should be pursued. Disease recurrence has been confirmed over a decade after the initial management [5], and there have been previous reports of early disease progression [4]. Disease recurrence and/or progression can be effectively assessed through the use of CT and [18F]FDG PET/CT, according to a previous report [4]. In this light, it is crucial to strictly follow up with patients with pediatric MSO for short-term and long-term outcomes.

## Conclusions

Physicians need to be aware of the possibility of MSO when assessing ovarian masses in pediatrics. When suspected, surgical and MDC consultation should be advocated for timely and optimal interventions. For locally confined diseases, our reported case proves that a thyroid and fertility-preserving approach is feasible. Through this approach, fertility preservation can be maintained for childbearing while pursuing routine and regular follow-up. However, such evidence is singular and short-term. Therefore, further knowledge of pediatric MSO is eagerly anticipated in the near future to better understand disease patterns and survival for such a challenging age group.
